# P-322. Providers Prepared and Eager to Integrate Biomedical HIV Prevention into Gender Affirming Care

**DOI:** 10.1093/ofid/ofaf695.541

**Published:** 2026-01-11

**Authors:** Nancy Aitcheson, Nadia Dowshen, Caroline O’Brien, Florence Momplaisir, Moira Kyweluk, M Grabill

**Affiliations:** University of Pennsylvania, Atlanta, Georgia; Children’s Hospital of Philadelphia, Philadelphia, Pennsylvania; Perelman School of Medicine, Philadelphia, Pennsylvania; University of Pennsylvania, Atlanta, Georgia; Plume Health, Philadelphia, Pennsylvania; Perelman School of Medicine, Philadelphia, Pennsylvania

## Abstract

**Background:**

Despite national progress in reducing HIV incidence, trans people in the United States remain disproportionately affected by HIV. Interdisciplinary research shows that receiving gender-affirming healthcare (GAHC) is linked to improved health outcomes. GAHC settings represent vital access points for addressing broader health needs, including biomedical HIV prevention. This study explores provider perspectives on co-locating biomedical HIV prevention and GAHC services.

**Methods:**

We recruited 20 geographically diverse, self-identified GAHC providers from a large, national GAHC telehealth company and a national GAHC provider listserv to complete semi-structured interviews about their comfort and experiences with offering both biomedical HIV prevention (Post Exposure Prohylaxis (PEP) and Pre-Exposure Prophylaxis (PrEP)) and GAHC, as well as perceived barriers and facilitators to co-locating these services. Interviews were transcribed verbatim and analyzed using grounded theory.

**Results:**

Providers expressed strong interest in and experience with offering both GAHC and biomedical HIV prevention. Oral PrEP was more commonly prescribed than injectable PrEP, due to insurance issues and provider hesitancy. Use of oral PrEP (versus injectable PrEP) was highlighted by both providers who saw clients in person and those who primarily practiced via telehealth. While less familiar with managing PEP, providers saw it as a useful complement to PrEP (e.g., “PEP to PrEP” and “PEP in Pocket” prescriptions).

Facilitators of co-located, integrated care included PrEP navigators, on-site or mail-order pharmacies, and trusting patient-provider relationships. Barriers included insurance logistics, outdated prescribing guidelines that fail to reflect trans people’s experiences (equating transgender women's indications for PrEP with those for men who have sex with men), and the challenge of coordinating additional or asynchronous bloodwork.

**Conclusion:**

Providers showed interest in and capacity for delivering biomedical HIV prevention within GAHC. Addressing logistical and systemic barriers to oral and injectable PrEP and PEP access, and co-locating and integrating these services into GAHC, may be a promising strategy to reduce HIV incidence among trans individuals.

**Disclosures:**

All Authors: No reported disclosures

